# Assessment of the medical expenditure of the basic health insurance in Morocco

**DOI:** 10.11604/pamj.2020.35.115.13076

**Published:** 2020-04-14

**Authors:** Amal Yassine, Abdelkader Jalil Hangouche, Naoufel El Malhouf, Siham Maarouf, Jamal Taoufik

**Affiliations:** 1 Laboratory of Therapeutic Chemistry, Faculty of Medicine and Pharmacy Rabat, Mohamed V University, Morocco; 2 National Agency of Health Insurance, Rabat, Morocco; 3 Laboratory of Physiology, Faculty of Medicine and Pharmacy of Rabat, Mohamed V University, Morocco

**Keywords:** Basic health insurance, medical expenditure, long-term disease, Morocco

## Abstract

**Introduction:**

The health care consumption for the population insured by the Basic Health Insurance in Morocco are paid directly to the care providers for the health care or health products from the health insurance funds. The level of expenditure recorded is changing at an accelerated rate than the financial resources. The objective of this study is to evaluate the health care consumption care by the insured population under the Basic Health Insurance.

**Methods:**

This is a cross-sectional study analysis of the economic data collected by the National Moroccan Health Insurance Agency Related to the expenditures from the health insurance fund for both public and private sectors to identify the behavior of the consumption of health care by the insured population under the Basic Health Insurance.

**Results:**

The medical expenditure of the covered population by the basic Health Insurance in Morocco has almost doubled from 354800 to 652500 US Dollars between 2009 and 2014 with significant increase in the public sector than the private sector. The share of expenditures in the public ambulatory care sector under Basic Health Insurance is higher relative to the hospital care. Although in the private sector the share of expenditures for both types of care varies. In 2014, the drug item expenditure accounted for 33% of Health Insurance expenses for both sectors. The level of health care consumption among the population in Long-Term Illness (LTI) represents 49,29% of the total expenditure by the Health Insurance whereas its insured covered population does not exceed 2,78%.

**Conclusion:**

Controlling the medical expenditure of the health insurance requires strengthening and the development of regulatory measures that contribute to the health reforms. For chronic diseases, it is necessary to put in place prevention actions.

## Introduction

According to the World Health Organization (WHO), any progress towards universal coverage must take into account three essential dimensions relating to the extension of medical coverage to the uncovered persons, reducing the insured participation in the costs of care and expanding health care by the health insurance [1]. In Morocco, these three dimensions constitute levers that determine the success of the universal medical coverage project. The principle of universality has been reinforced in the new constitution of 2011 in its article 31 that stipulates the right of all Moroccan citizens to the Medical Coverage [2]. A successful health financing system should aim to generate sufficient and sustainable resources, to make optimal use of resources and to ensure financial accessibility for all to health care [3]. A health financing system has the following functions [1]: 1) the collection of resources, a process that allows a health system to receive money from households, businesses, government and other organizations including donors. 2) The pooling of health care payment risks by the accumulation and the management of the revenues in order to spread the health care payment on the insured group rather on the individuals. 3) Purchasing is a process by which contributions are used to pay health providers to deliver a set of specific or non-specific health interventions. The purchase can be either passive or strategic: the passive purchase being realized according to the predetermined budgets or by the payment of the invoices when they presented. However, the strategic purchase implies a continuous search for the best health services. In Morocco, Law n° 65-00 on Basic Medical Coverage establishes the Basic Compulsory Health Insurance that came into effect in 2005. This coverage started first, by employees in the public and private sectors, to gradually extend to students in 2016 and then to the self-employed in 2017. For vulnerable and poor people there is another system as system called “Medical Assistance Scheme for economically deprived persons” that started in 2012 [4]. The management of the Basic Compulsory Health Insurance is entrusted to two health insurance funds: 1) the National Fund of Social Welfare Organizations for Public Sector Employees and Students; 2) the National Social Security Fund for employees in the private sector and self-employed. The National Health Insurance Agency is a regulatory institution that is in charge of the technical supervision and regulation of health insurance (Quality of services and control of expenses) [4]. In Morocco, the health care consumption for the population insured by the Basic Health Insurance are paid directly to the care providers for the health care or health products from the health insurance funds. The medical expenses of the Basic Health Insurance are generated on the one hand by the list of treatment or care covered and predefined in the provisions of Law no°65-00 on the basic medical cover code [4] and on the other hand by the rate medical benefits coverage predefined by application decrees [5,6] for each sector of activity (public or private). This reimbursement rate increases in the case of serious or disabling Long-Term Illness (LTI) for both sectors [7]. The tariff of medical services is set out at the establishment of the national agreements with the various healthcare providers. The terms of the agreements are set out in Articles 18 to 25 of Law n ° 65-00 [4] on the Code of Basic Medical Coverage. These prices are either in the form of a National Reference Tariff or in the form of a suite price for the treatment care required in addition to the technical medical and other indirect costs [7]. The level of expenditure recorded is changing at an accelerated rate than the financial resources. This requires the strengthening and the development of the regulatory measures. This requires a prior knowledge of the insured health care consumption profile, as well as the opportunities available to control the expenditure in the context of health insurance. However, monitoring the budget balance, sustainability and continuity of the medical insurance structure is equally important. The objective of this study is to evaluate the health care consumption of the population covered by Basic Health Insurance in Morocco.

## Methods

**Type of study**: this is a cross-sectional study analysis of the economic data to identify the behavior of the health care consumption of the insured persons by the Basic Health Insurance.

**Source of the study**: the expenditures data were collected from the Moroccan National Health Insurance Agency for both public and private sectors between 2009 and 2014. This data collection is done after each accounting year of the health insurance funds from 2008 to 2014. The collection of data was done in compliance with the regulatory provisions of Law no° 09-08 [8] on the protection of individuals with regard to the processing of personal data. The data includes only the economic variants to be studied.

**Research structure**: Moroccan National Health Insurance Agency and the Faculty of Medicine of Rabat, Morocco.

**Criteria for inclusion and exclusion**: the exploitation of the identified parameters results from the exercise of the health insurance funds between the period between 2008 and 2014. Only the data validated by the board of directors of the National Health Insurance Agency have been exploited. The study of expenditures stopped in 2014 and does not include student and self-employed expenses. Beneficiaries of the medical assistance scheme for the economically deprived population are not included. They depend on another system. The financial statements and the management procedures of the health insurance funds were excluded in this study.

**The studied parameters**: 1) medical expenditure in Health insure for both sectors (public and private); 2) expenses according to the nature of the acts (ambulatory care or hospitalization) 3) expenses by Insured Profile: a) employees versus pensioner with their beneficiaries; b) Population with Long Term Illness (LTI). 4) the medical expense by medical act.

**Note that**: 1) the employee is defined as a person in professional activity with his beneficiaries (spouse (s) and children); 2) the pensioner is defined as a retired person with his dependents. Pensioners include retired or disabled persons, surviving spouse (s), and dependents (children); 3) for Ambulatory care: the insured person advances the costs of care and the health insurance fund reimburses within a period not exceeding three months. 4) for Hospitalization: the health insurance fund pays the health professionals directly and the insured pays the co-payment only. 5) the population with a Long-term Illness (LTI) is all the beneficiaries registered with the health insurance funds for at least one chronic pathology.

**Limitations of the study**: the process of affiliation to health insurance is a very dynamic process. The evolution results of the study parameters were static and didn't take into account the continuous dynamics of the population covered between each year so the result figures represent amounts paid during the financial year regardless of the date of occurrence of medical care.

## Results

**Figure 1:**
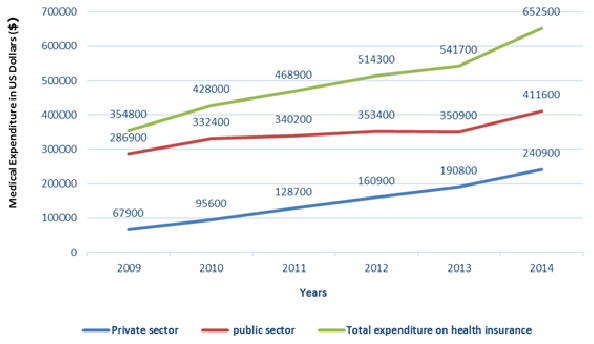
Evolution of the medical expenditure of Health Insurance between 2009 and 2014 in thousands of US Dollars ($)

**Figure 2:**
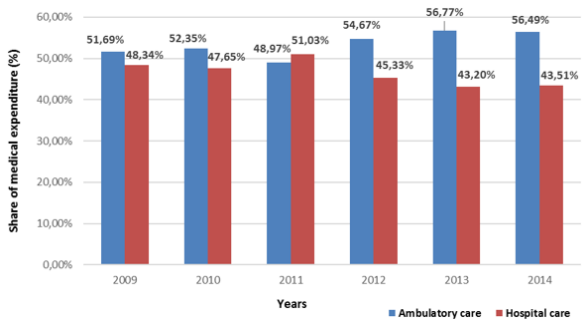
Evolution of the proportion of public sector medical expenditure according to the nature of the act between 2009 and 2014

**Figure 3:**
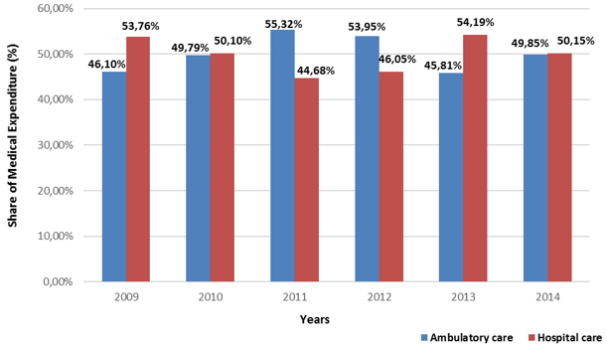
Evolution of the proportion of private sector medical expenditure according to the nature of the act between 2009 and 2014

**Figure 4:**
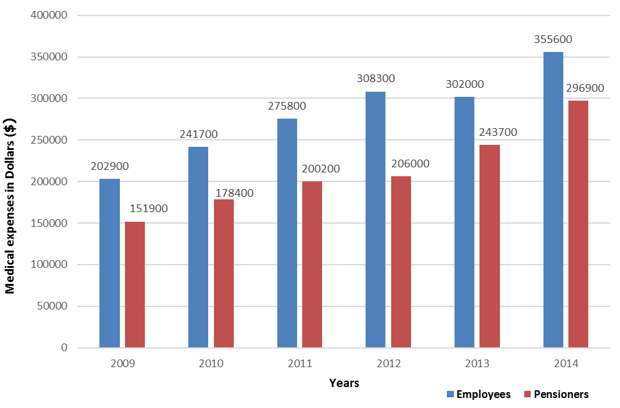
Evolution of health insurance medical expenses in thousands of US dollars ($) according to the type of insured between 2009 and 2014

**Figure 5:**
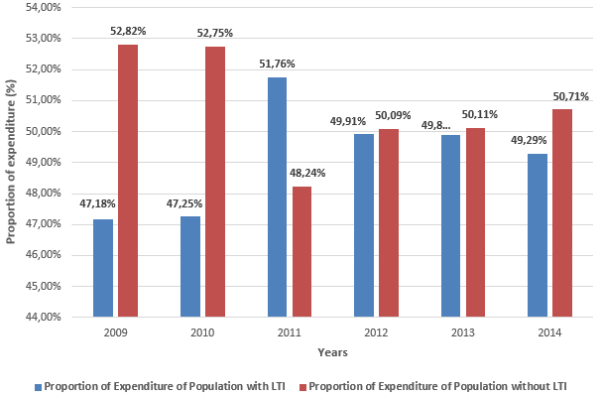
Evolution of the proportion of medical expenses between the population with Long Term Illness (LTI) and population without LTI between 2009 and 2014

The medical expenditure of the covered population by the basic Health Insurance in Morocco has almost doubled from 354800 to 652500 US Dollars between 2009 and 2014 with significant increase in the public sector than the private sector (Figure 1). The share of expenditures in the public ambulatory care sector under Basic Health Insurance is higher relative to the hospital care (Figure 2). Although in the private sector the share of expenditures for both types of care varies (Figure 3). The growth rate of medical expenses of pensioners is much more significant than in employees (12.39% vs 10, 39%) (Figure 4). In 2014, The drug costs recorded a 33% share of the expenditure by the Moroccan Basic Health Insurance for both sectors followed by hospitalization (16%) for the public sector and dialysis (16%) for the private sector respectively [9]. The level of health care consumption by the Long-Term Illness (LTI) patients represents a 49.29% of the total of health insurance expenditure in 2014 (Figure 5). In health Insurance, diseases with high medical expenditure are respectively Chronic Renal failure (29.4%), Malignant Tumors (21.2%), Severe Hypertension (11.9%) And Diabetes (10.2%) [9].

## Discussion

The Basic Medical Coverage faces a number of challenges. First, the high cost access to innovative medical quality care, additional to ensuring the sustainability of medical coverage and to guarantee the financial balance of the health insurance funds. Second, the inequality of the care offered throughout the Moroccan territory. Finally, the progression of non-communicable diseases (chronic diseases) linked to the change in the epidemiological profile of the Moroccan population. The control of health expenditure requires a better understanding of the patient's behavior of health care consumption and costs to the Basic Health Insurance and the determination of the key items of medical expenditure [10]. The evolution of the medical expenditure of the Basic Health Insurance has increased between 2009 and 2014 for both public and private sectors. This development follows the evolution of the population covered in both sectors [9]. The large share of public sector expenditure is explained by the higher coverage rate of medical services than the private sector. In the public sector, the significant difference between ambulatory care and the hospital care is due to the use of ambulatory care much more than hospital by the insured persons. Although, in the private sector, this difference between the two types of care is due to the exclusion the outpatient care before 2009.

Since the start of health insurance in 2005, outpatient care was not eligible for reimbursements in the private sector including persons carrying chronic diseases. However, changes to the refund structure commenced in 2009, the behavior of insured persons regarding the refund application files did not automatically follow this dynamic. Then, the National Social Security Fund (private sector fund) started to exonerate the co-payment for Long-Term Illness (LTI) according to the type of the disease. This exemption covers all acts related to Long-Term Illness (LTI) by applying the coverage rate for each disease ranging from 77 to 100% [11]. Medical expenditure is higher in the pensioners covered population, while the number insured is low, compared to employees [9]. The rate of growth in consumption is probably due to the predominance of the population with chronic disease. In 2014, Drug costs showed a high share of 33% of expenditure to the Health Insurance for both sectors. Studying this parameter may assist in the reduction to the high level of drug cost to the system and may ensure the balance of the health insurance funds.

In the end of 2014, the population suffering from Chronic Diseases has a very low share not exceeding 3% of the total population covered by the basic health insurance [9] whereas 25% Moroccan population are affected by Chronic Illnesses [12]. This covered population cost half of the resources of the health insurance funds. This medical expenditure is explained by the high reimbursement rate for persons with LTI and in most cases have more than one chronic disease. This situation will cause the patient to use several medical services at the same time, which entails more expenses for the Health Insurance. The increase in expenditure for chronic diseases is due to the prevalence of diseases in the population covered [12]. Knowledge of the expenditure portion for each disease would strengthen the preventive actions and develop adapted therapeutic protocols.

## Conclusion

The performance of the basic medical coverage system in Morocco requires identification of the variants that comprise it, understanding the profile and the behavior of the health care consumption of the population covered. Controlling the medical expenditure of health insurance requires the development, and the strengthening of regulatory measures that contributes to the health reforms. For chronic diseases, it is necessary to put in place prevention actions.

### What is known about this topic

- The entry of the Basic Health Insurance in Morocco;
- The Financing of Health in Morocco.

### What this study adds

- The evolution of the medical expenses of the Basic Health Insurance in Morocco;
- Determination of the health care consumption profile of the population covered by the Health Insurance in Morocco.

## Competing interests

The authors declare no competing of interest.
